# Activation State-Dependent Substrate Gating in Ca^2+^/Calmodulin-Dependent Protein Kinase II

**DOI:** 10.1155/2017/9601046

**Published:** 2017-12-17

**Authors:** D. E. Johnson, A. Hudmon

**Affiliations:** Biochemistry and Molecular Biology, Stark Neuroscience Research Institute, Indiana University School of Medicine, Indianapolis, IN 46202, USA

## Abstract

Calcium/calmodulin-dependent protein kinase II (CaMKII) is highly concentrated in the brain where its activation by the Ca^2+^ sensor CaM, multivalent structure, and complex autoregulatory features make it an ideal translator of Ca^2+^ signals created by different patterns of neuronal activity. We provide direct evidence that graded levels of kinase activity and extent of T^287^ (T^286^* α* isoform) autophosphorylation drive changes in catalytic output and substrate selectivity. The catalytic domains of CaMKII phosphorylate purified PSDs much more effectively when tethered together in the holoenzyme versus individual subunits. Using multisubstrate SPOT arrays, high-affinity substrates are preferentially phosphorylated with limited subunit activity per holoenzyme, whereas multiple subunits or maximal subunit activation is required for intermediate- and low-affinity, weak substrates, respectively. Using a monomeric form of CaMKII to control T^287^ autophosphorylation, we demonstrate that increased Ca^2+^/CaM-dependent activity for all substrates tested, with the extent of weak, low-affinity substrate phosphorylation governed by the extent of T^287^ autophosphorylation. Our data suggest T^287^ autophosphorylation regulates substrate gating, an intrinsic property of the catalytic domain, which is amplified within the multivalent architecture of the CaMKII holoenzyme.

## 1. Introduction

Long-term potentiation (LTP) is a long-lasting enhancement of excitatory postsynaptic currents that many believe to be a cellular correlate of learning and memory [1]. While LTP can be observed in multiple areas of the mammalian brain (as well as in lower organisms), the Schaffer collateral/CA3 synapses onto CA1 pyramidal neurons of the hippocampus are a classic example whereby brief patterns of high-frequency activity favor LTP to enhance neuronal connectivity [2]. The opposing change in synaptic plasticity, long-term depression (LTD), is produced by low firing frequencies [2]. As might be expected, activity-dependent forms of plasticity like LTP and LTD both require the second messenger calcium (Ca^2+^). While LTP has been historically ascribed to kinase activity and LTD to dephosphorylation and phosphatase activity [3], a common effector system capable of decoding Ca^2+^ spike frequency into different functional outputs would be strategically positioned to regulate both LTP and LTD. The Ca^2+^/calmodulin- (CaM-) dependent protein kinase II (CaMKII) is an ideal regulator of synaptic plasticity because it has complex autoregulatory features that make it ideally suited for regulating activity-dependent plasticity [4–7].

**Figure 1:**
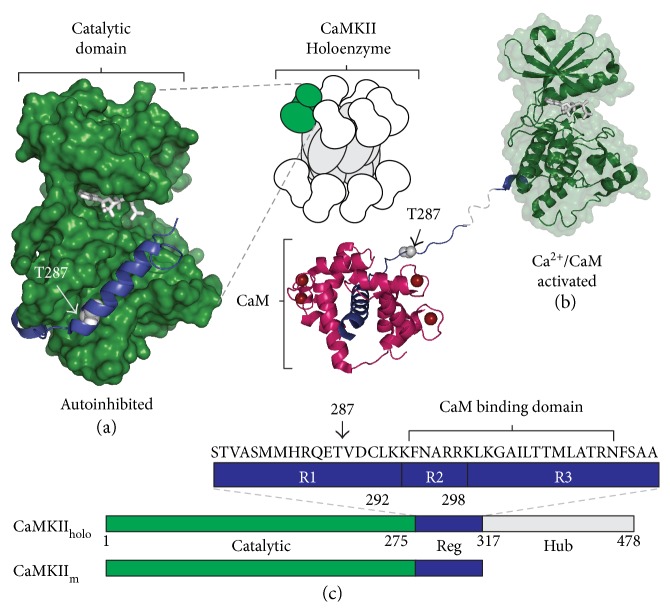
Molecular structure and schematic models for CaMKII autoregulation and activation. (a–c) Structural representations of CaMKII's catalytic domain (green), autoregulatory domain (ARD) (blue), and hub domain (gray). (a) Illustration of CaMKII holoenzyme with detailed view of the catalytic domain. Adapted X-ray crystal structure shows monomeric human CaMKII*δ*_11–309_ (PDB ID: 2VN9; [11]) in the autoinhibited state with the ARD (blue) and modified to include ATP in the active site (PDB ID: 1ATP; [71]). (b) hCaMKII*δ*_11–335_ activated (PDB ID: 2WEL; [11]) by Ca^2+^ (red spheres) and calmodulin (CaM) (magenta) and exposing T^287^ (white) for autophosphorylation. (c) Linear schematic of CaMKII holoenzyme (CaMKII_holo_) and monomeric CaMKII (CaMKII_m_, or CaMKII_1–317_). Expanded view shows the ARD (blue) with subdivisions R1 (T^287^ containing region) and R2/R3 (CaM-binding region) [72].

In mammals, CaMKII family is composed of four closely related isoforms (*α*, *β*, *γ*, and *δ*) where it regulates diverse substrates in cellular processes ranging from metabolism and cell cycle control to Ca^2+^ homoeostasis and excitability [8]. Thus, like other multifunctional serine/threonine protein kinases (e.g., PKA and PKC), CaMKII phosphorylates many different cellular substrates in different subcellular compartments to globally coordinate cellular function. CaM is a ubiquitous Ca^2+^ sensor that activates CaMKII by binding to a target domain that disinhibits the autoregulatory domain (ARD) to allow substrate binding to the catalytic surface (Figures 1(a), 1(b), and 1(c)).

The unique multivalent structure of CaMKII permits complex forms of autoregulation [9, 10]. The CaMKII holoenzyme is comprised of 12–14 subunits [11–13] that permits coincident CaM binding to support an intraholoenzyme-intersubunit autophosphorylation [14, 15] (T^286^* α* isoform and T^287^* β*, *γ*, and *δ* isoforms) reaction (for convenience, we use T^287^ throughout the paper). While CaMKII is known to undergo autophosphorylation on multiple sites [16], many of which have unknown functions, the T^287^ autophosphorylation site within the ARD leads to enhanced CaM binding affinity in the presence of Ca^2+^ [17] and Ca^2+^/CaM-independent activity (autonomous) in low Ca^2+^ [18, 19]. The unique structure and autoregulation make CaMKII an ideal sensor to encode Ca^2+^-spike frequency into T^287^ autophosphorylation; a process influenced by Ca^2+^-spike duration and amplitude [20], phosphatase activity [21], alternative spliced linker length, and CaM availability [22, 23].

While CaMKII is present throughout the body, it is highly enriched at neuronal connections. In fact, CaMKII is one of the most abundant proteins in the postsynaptic density (PSD) [24–26], a cytoskeletal organelle located just underneath the spine plasma membrane that localizes the postsynaptic signaling machinery with presynaptic release sites. While CaMKII appears to have multiple functions in synaptic plasticity, phosphorylation of GluA1 receptors to regulate their trafficking and signaling appears to be critical for LTP [7]. CaMKII's role in LTP and Ca^2+^ signaling in the postsynaptic compartment is also linked to its ability to undergo translocation to NMDA receptors and undergo autophosphorylation [5, 27, 28], processes that might serve to restrict the kinase to its targets within the PSD. However, like the second messenger Ca^2+^, CaMKII is also implicated in LTD [29, 30]. And while the role of CaMKII in postsynaptic LTD has not been as intensively studied as its role in LTP [4], CaMKII was recently shown to differentially phosphorylate two different phosphoacceptor sites on GluA1 in LTP versus LTD [31]. Because LTP is promoted by strong stimuli versus weak for LTD, one plausible explanation is that graded levels of CaMKII activity and/or autophosphorylation promote differential substrate phosphorylation to induce the opposing forms of synaptic plasticity. However, recent studies have revealed little or no impact of autophosphorylation on the catalytic output of CaMKII [32, 33]. Using purified PSDs and immobilized peptide arrays to create a well-defined multisubstrate system, we test the hypothesis that the catalytic output and substrate selectivity of CaMKII are regulated by its activation state. Our data support a model whereby T^287^ autophosphorylation regulates substrate gating, an intrinsic property of the catalytic domain, which is amplified within the multivalent architecture of the CaMKII holoenzyme.

## 2. Results

CaMKII translocation and phosphorylation of PSD proteins are believed to play an important role in postsynaptic modifications supporting LTP [4–7]. We determined whether PSD phosphorylation by CaMKII induced by saturating Ca^2+^/CaM is *equivalent* or *different* to monomeric CaMKII catalytic activity. Conceptually, one might expect that a similar number of activated T^287^ autophosphorylated subunits (T^286^ in alpha isoform) would produce similar levels of substrate phosphorylation within the PSD regardless of whether the catalytic subunits are within a holoenzyme or monomeric. While all four CaMKII isoforms exhibit a high degree of sequence identity in their catalytic and regulatory domains, we selected the human *δ*CaMKII isoform for its enhanced stability in our kinase assays. While the association domain hub was deleted from monomeric CaMKII (1-317) (Figures 1(a), 1(b), and 1(c)), this recombinant kinase, like the native holoenzyme, retains its Ca^2+^/CaM dependence and undergoes T^287^ autophosphorylation as an intersubunit reaction mechanism which we accomplished in a prereaction (see Materials and Methods). Postsynaptic densities were acquired from adult rats (male and female Sprague-Dawley) using differential centrifugation and detergent extraction to obtain the PSD Triton-insoluble fraction (see Materials and Methods) [34, 35]. The PSD preparation displayed a characteristic size and ultrastructure (data not shown) and SDS-PAGE separation and a Coomassie blue staining revealed the complex protein profile characteristic of PSD preparations.

Our intent was to focus on characterizing PSD phosphorylation from exogenously added CaMKII; therefore, several steps were taken to accomplish this. First, rat brains were extracted and snap frozen in liquid nitrogen as rapidly as possible (~90 secs) after animal sacrifice to reduce CaMKII translocation to the PSDs in response to decapitation [36]. Second, to minimize autonomous activity of endogenous PSD CaMKII, isolated PSDs were dephosphorylated overnight using a PP1*α* catalytic fragment. The 6X-His-tagged phosphatase was removed the next day using immobilized metal affinity chromatography. Third, the allosteric small molecule CaMKII inhibitor, KN93, was included in the PSD phosphorylation reaction to inhibit endogenous CaMKII from undergoing T^287^ autophosphorylation with addition of Ca^2+^/CaM (as KN93 prevents Ca^2+^/CaM binding/activation but does not inhibit activated T^287^ autophosphorylated CaMKII). Fourth, phosphatase and protease inhibitor cocktails were used in the PSD phosphorylation reactions to disrupt dephosphorylation and proteolysis reactions. Finally, exogenous CaMKII was preautophosphorylated at T^287^ with thiol-ATP (ATP*γ*S) to limit potential dephosphorylation and to minimize subsequent incorporation of ^32^P in exogenous CaMKII autophosphorylation sites.

**Figure 2:**
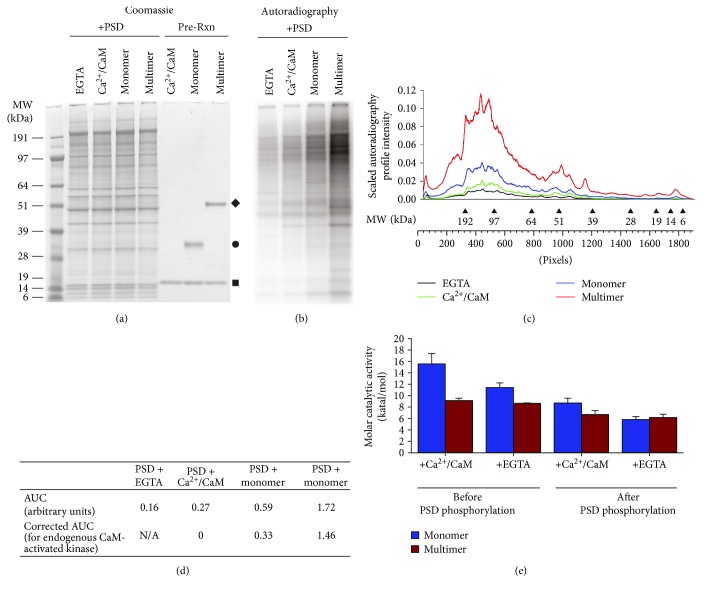
Maximally active CaMKII holoenzymes enhance the phosphorylation of PSD proteins over monomeric CaMKII. (a–d) Isolated rat PSDs were phosphorylated in low (EGTA) or high calcium (Ca^2+^/CaM) to assess endogenous, copurified kinases. PSDs were also phosphorylated with the addition of equal molar catalytic subunits of T^287^ autophosphorylated CaMKII multimer (CaMKII_holo_^+P^ = diamond) or monomer (CaMKII_m_^+P^ = circle). (a) Coomassie-stained gel illustrating the PSD profile and the purified proteins used in the reaction. Square indicates CaM. (b) Phosphorimaging of the SDS-PAGE showing ^32^P-phosphoprotein bands. (c) Phosphorylation profile scaled to total Coomassie-stained protein. Molecular weight markers indicated along the scaled axis (pixels) representing the scaled lane of the SDS-PAGE. (d) Total phosphorylation for each condition assessed using the area under the curve (AUC) as arbitrary intensity units. (e) Quantitation of Ca^2+^-dependent (Ca^2+^/CaM) and Ca^2+^-independent (EGTA) enzymatic activity (katal/mol) of CaMKII measured via soluble peptide assays with radioactive (*γ*^32^P) ATP. CaMKII, monomer or multimer, was autophosphorylated at T^287^ prior to the PSD phosphorylation (and activity measurement) (see Materials and Methods) (*n* = 3). Error bars denote ± s.d.

Postsynaptic densities were phosphorylated (plus [*γ*-^32^P-] ATP) with T^287^ autophosphorylated holoenzyme (CaMKII_holo_^+P^) or monomer (CaMKII_m_^+P^) in the presence of Ca^2+^/CaM. Similar levels of catalytic activity were applied for both forms of the autophosphorylated CaMKII (see Materials and Methods). To control for endogenous Ca^2+^/CaM-dependent or Ca^2+^/CaM-independent kinase activity, PSDs were also phosphorylated in the presence of Ca^2+^/CaM or EGTA, respectively. As described in detail in Materials and Methods, PSD phosphorylation was quantified by phosphorimaging of the ^32^P incorporation following SDS-PAGE separation of the proteins (Figures 2(a) and 2(b)). The corresponding phosphorylation data (autoradiography of ^32^P incorporated bands) (Figures 2(c) and 2(d)) was scaled to the total PSD protein in a given sample based on the area under the curve (AUC) as described in Materials and Methods. Under these conditions, the addition of Ca^2+^/CaM alone produced less than a 2-fold increase in the total phosphorylation compared to other Ca^2+^/CaM-independent kinases (the EGTA condition; Figure 2(d)). The inclusion of exogenous CaMKII_m_^+P^ produced ~2-fold increase in the phosphorylation compared to Ca^2+^/CaM-activated PSDs alone, suggesting that PSD substrates were available to exogenously added kinase (Figure 2(d)). The CaMKII_holo_^+P^ produced a 6.4-fold increase in phosphorylation compared to Ca^2+^/CaM-activated PSDs (Figure 2(d)). More importantly, compared to the T^287^ autophosphorylated monomer, there was a 4.4-fold increase in total phosphorylation when corrected for endogenous Ca^2+^/CaM-stimulated phosphorylation (Figure 2(d)) even though the holoenzyme might be expected to have less accessibility to substrates in the PSD compared to the monomer because of their size difference (~750 kDa versus 35 kDa, resp.).

The amount of CaMKII enzymatic activity (both Ca^2+^/CaM stimulated and autonomous) added to the PSD phosphorylation reaction was determined both before and after the experiment via a soluble kinase assay using the CaMKII-specific peptide AC-2 (see Materials and Methods). While the catalytic activity of the monomeric kinase was greater than the holoenzyme at the initiation of the PSD phosphorylation reaction, the level of activity at the end of the PSD phosphorylation was similar between monomeric and multimeric CaMKII (Figure 2(e)). While it is unclear why a loss of monomeric activity is observed at the end of the PSD reaction, no loss of enzymatic activity is observed for either CaMKII_holo_^+P^ or CaMKII_m_^+P^ in the absence of PSD protein (data not shown). Thus, while the enzymatic activity of monomeric CaMKII is favored or equal (start or end of the reaction) to that of the holoenzyme CaMKII, substrate phosphorylation is higher for CaMKII when multiple subunits are activated within the holoenzyme for a diffusion-restricted environment like the PSD.

Specificity for the switch-like behavior in CaMKII critical for LTP [3] may arise from this enzyme's ability to translate Ca^2+^-spike frequency produced by brief patterns of high-frequency stimulation into graded levels of activity and T^287^ autophosphorylation [20]. Thus, we tested whether regulating the extent of activation within the CaMKII holoenzyme regulates substrate selectivity. However, endogenous effectors, scaffolding, and other regulatory proteins within the PSD could complicate our analysis; therefore, we created a multiple substrate SPOT array in order to explore how graded levels of CaMKII activity influence substrate utilization (see Materials and Methods). An obvious advantage of this immobilized peptide assay is that diverse substrates can be exposed to identical conditions without high-affinity substrates dominating mixed soluble substrate reactions [37]. We selected four well-studied CaMKII substrates studied with varying affinities as determined by standard solution kinetics—a high-affinity substrate (GluN2B_S1303_, *K*_m_ = 4.6 ± 1.1 *μ*M) and three lower-affinity, weak substrates (Syntide, *K*_m_ = 43.5 ± 2.3 *μ*M; Vimentin_S83_ and GluA1_S849_, *K*_m_ > 1 mM). All peptide sequences are denoted in Materials and Methods.

**Figure 3:**
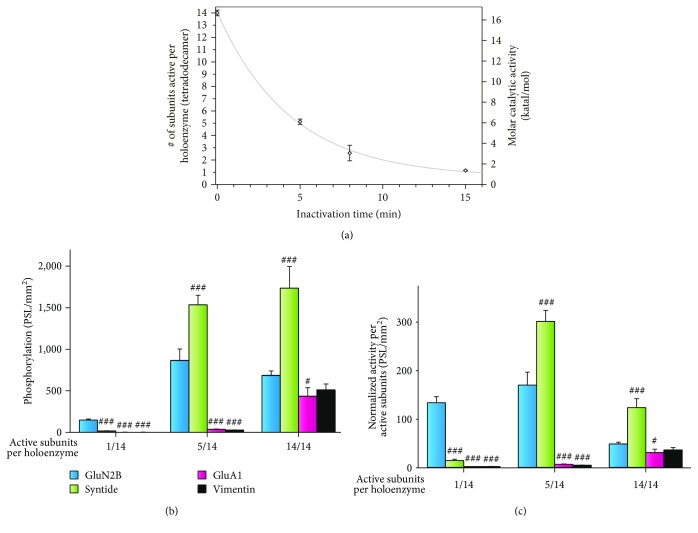
Enhanced phosphorylation of intermediate and weak SPOT substrates with increasing number of active subunits per holoenzyme. (a) Time-dependent inactivation of CaMKII by basal autophosphorylation. Ca^2+^/CaM-stimulated activity of CaMKII_holo_ expressed in molar terms (katal/mol) and measured via standard soluble peptide (AC-2) assays with radioactive (*γ*^32^P) ATP. Enzymatic activity was measured following preinactivation reactions of various times (see Materials and Methods); mean ± s.d. (*n* = 3). By calculating the average enzymatic activity per subunit in a fully activatable tetradecameric holoenzyme (i.e., 0 min of inactivation), the average number of activated subunits per holoenzyme was determined for each condition. (b, c) Phosphorylation ([^32^P] phosphate incorporation) of immobilized CaMKII substrates (GluN2B, Syntide, GluA1, and Vimentin synthesized via SPOTs) by CaMKII_holo_ exhibiting 1, 5, or 14 activated subunits per holoenzyme. Substrates ranked from the lowest to the highest *K*_m_. (b) Raw SPOT phosphorylation (PSL/mm^2^) represented as mean ± s.d. (*n* = 3). (c) SPOT phosphorylation normalized by the number of subunits activated per holoenzyme and represented as mean ± s.d. (*n* = 3). One-way ANOVA of log-normalized data with Holm-Šídák posttest compared to GluN2B within each group (^#^*P* < 0.05, ^###^*P* < 0.001).

To create graded levels of CaMKII activity and T^286^ autophosphorylation in conditions of saturating Ca^2+^/CaM, we took advantage of the basal, inhibitory (Ca^2+^/CaM-independent) autophosphorylation (i.e., capping) described previously [38]. Specifically, naïve multimeric CaMKII is exposed to Mg^2+^/ATP for varying times in the absence of Ca^2+^/CaM. This is a relatively slow intramolecular autophosphorylation reaction (complete in 15–20 mins) within the CaM-binding domain (T^306^ in CaMKII*δ*) that blocks subsequent Ca^2+^/CaM binding/activation [19, 38–40]. The degree of inactivation is measured by exposing the Mg^2+^/ATP-treated CaMKII to saturating Ca^2+^/CaM and AC-2 in a soluble kinase assay (see Materials and Methods). We expressed maximal catalytic activity as a function of the number of subunits in the holoenzyme (the human CaMKIIδ holoenzyme is tetradecameric [11]). This allowed for the determination of the molar catalytic activity associated with one catalytic subunit. Using this metric, the average number of activatable subunits per holoenzyme was calculated for each inactivation timepoint shown in Figure 3(a) (see Materials and Methods). Thus, by normalizing our treated or “capped” holoenzymes to naïve untreated CaMKII (14/14 subunits), we calculated that residual bulk kinase activity in solution as represented on average by holoenzymes with 1/14 or 5/14 subunits is available for Ca^2+^/CaM activation.

Using this capping pretreatment strategy to limit the number of Ca^2+^/CaM activatable subunits per holoenzyme, we observed that one active subunit per holoenzyme (CaMKII_holo_^1/14^) displays preferential phosphorylation for the high-affinity substrate GluN2B (Figure 3(b)). In Figure 3(c), the phosphorylation data was normalized to account for the different levels of “starting” kinase activity produced by the capping reaction in order to better evaluate substrate selectivity profiles. Unlike GluN2B, increasing the number of activatable subunits per holoenzyme significantly increases relative phosphorylation of Syntide (intermediate substrate affinity) and GluA1 and Vimentin (weak substrates) (Figure 3(c)). While minimal changes in GluN2B phosphorylation were observed, a 20-fold increase in Syntide phosphorylation by submaximally activated CaMKII_holo_^5/14^ was measured (Figure 3(c)). Maximally activated CaMKII_holo_^14/14^ produced the greatest increase (12-fold) in phosphorylation of the weakest substrates, GluA1 and Vimentin (Figure 3(c)). These data indicate that intermediate- and low-affinity substrates require multiple activated subunits within the holoenzyme for phosphorylation. In contrast, high-affinity substrates are preferentially phosphorylated when the number of subunits per holoenzyme is limited. The reduced phosphorylation of high-affinity substrates seen in Figure 3(c) may result from substrates like NR2B possessing T-Site (i.e., targeting mode) characteristics [41], which allow substrates mimicking the ARD of CaMKII to bind in a mode that is biochemically distinct from the substrate binding pocket [42]. These stable, high-affinity interactions may punish substrate utilization via avidity effects as discussed further below.

**Figure 4:**
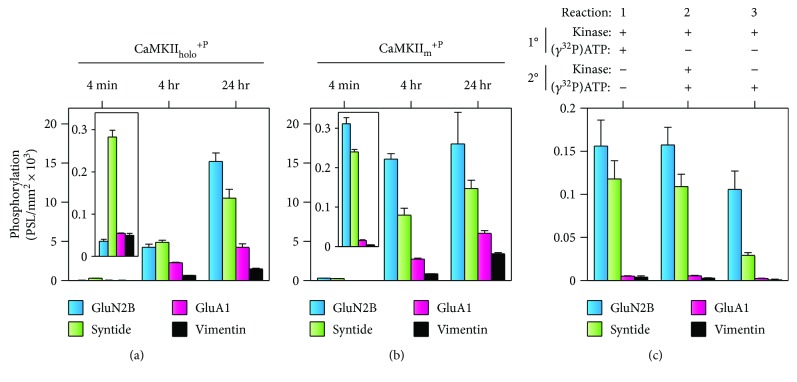
Substrate availability and reaction linearity for SPOT phosphorylation reactions. (a–c) Substrate phosphorylation profiles ([^32^P] phosphate incorporation of SPOT peptides). Phosphorylation time course of CaMKII_holo_^+P^ (a) or CaMKII_m_^+P^ (b) with an expanded view of 4 min reaction (inset); mean ± s.d. (*n* = 3). (c) Phosphorylation by CaMKII_m_^+P^ comparing a normal 4 min SPOT reaction (c-1) to reactions with only radioactive secondary 4 min reactions with (c-2) or without (c-3) additional kinase in the secondary reaction; mean ± s.d. (*n* = 3).

We elected to use the SPOTs immobilized peptide approach as it has the obvious advantage that multiple defined substrates can be examined simultaneously without having the high-affinity substrates dominate the diffusion-limited solution assay. However, it is still important for these interfacial reactions to obey specific requirements for any enzyme analysis, including a nonsaturating reaction (Figure 4). We observed that less than 2% of the available peptide for each substrate (as compared to a 24-hour reaction for GluN2B, Syntide, GluA1, and Vimentin) was phosphorylated by CaMKII_holo_^+P^ in our standard reactions (Figure 4(a)). While we did not extend the phosphorylation reactions longer than 24 hrs, this time point is likely at equilibrium as the extent of phosphorylation follows an expected ranking based on substrate affinity (GluN2B > Syntide > GluA1/Vimentin). Furthermore, we observed minimal differences between maximally activated (14/14 subunits) holoenzymes at 60 nM versus 5 nM (Figures 3(b) and 4(a)—inset, resp.), indicating that while the extent of phosphorylation is sensitive to kinase concentration, changes in the substrate phosphorylation profile are not. Thus, for our standard SPOT reactions (4 mins), changes in the phosphorylation profile are not due to differential substrate availability.

One caveat of using SPOT arrays for enzyme analysis is that they can be negatively influenced by avidity effects created by multivalent contacts [43]. For example, the binding affinity of a protein with its immobilized peptide target increased by over 1000-fold when a dimeric protein is compared to a monomeric variant [44]. Consistent with avidity effects contaminating CaMKII phosphorylation of the high-affinity substrates in the SPOT assays, we observed that phosphorylation for the high- (GluN2B) and intermediate-affinity (Syntide) substrates was suppressed as the number of activated subunits within the holoenzyme increased from 5/14 to 14/14. This becomes readily apparent when CaMKII substrate phosphorylation is normalized to the number of activated subunits in the capping experiments (Figure 3(c)). This avidity effect was not seen with monomeric CaMKII, as substrate phosphorylation profile for CaMKII_m_^+P^ follows the expected profile (i.e., higher affinity, greater substrate phosphorylation (Figure 4(b))). Again, linearity for the monomer CaMKII reaction rates was supported by the observation that in our standard SPOT reactions, less than 2% of the available peptide for each substrate (as compared to a 24-hour reaction for GluN2B, Syntide, GluA1, and Vimentin) was phosphorylated by CaMKII_m_^+P^ (Figure 4(b)). Conclusive support for a linear kinase-substrate reaction is supported by a pulse-chase style reaction in which nearly identical levels of ^32^P incorporation are obtained when a secondary reaction occurs on the same SPOT membrane immediately after an initial nonradioactive reaction (Figure 4(c)—reaction 1 versus 2). A modification of this assay whereby CaMKII_m_^+P^ is applied to the SPOT membrane in the absence of ATP^32^ reveals that bound kinase continues to form product over time when the membrane is washed and ATP^32^ is added during the chase without additional kinase (Figure 3(c)). In total, our standard SPOT reactions are not limited by substrate availability nor are they outside of a linear reaction range. However, because the reduced substrate phosphorylation of higher affinity substrates with maximally activated CaMKII_holo_^+P^ is likely contaminated by multivalent avidity effects, we employed the monomeric kinase to directly evaluate the contribution of T^287^ autophosphorylation on substrate selectivity.

**Figure 5:**
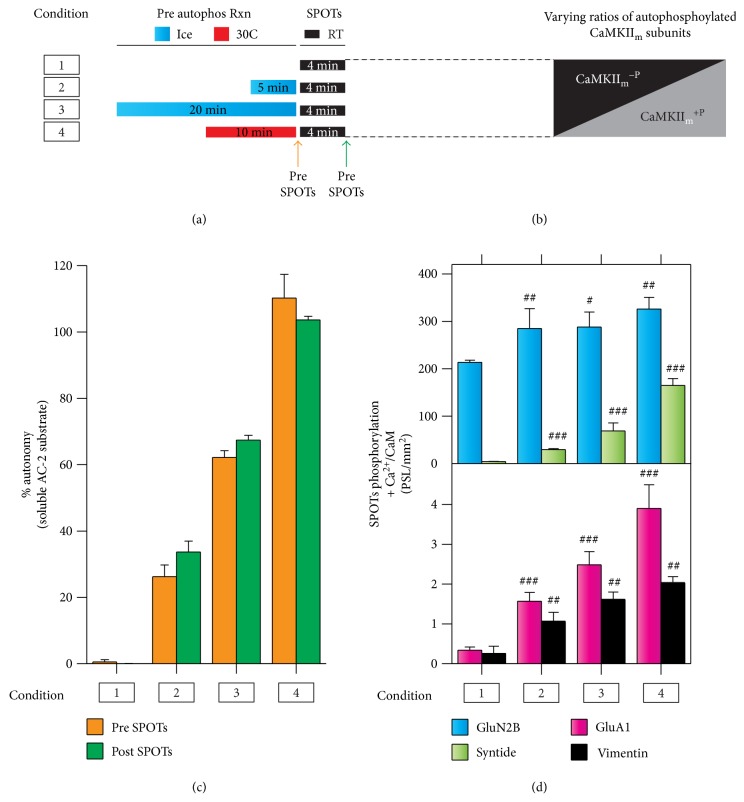
CaMKII substrate utilization and selectivity are differentially altered by T^287^ autophosphorylation. (a) Schematic representation of various conditions used to preautophosphorylate CaMKII_m_ prior to Ca^2+^/CaM-stimulated substrate phosphorylation reactions on SPOT arrays (see Materials and Methods). Essentially, time and/or temperature was altered to generate different extents of T^287^ autophosphorylation (i.e., varying ratios of CaMKII_m_^−P^/CaMKII_m_^+P^) as graphically illustrated in (b). (c) As a readout of T^287^ autophosphorylation, the % autonomy (i.e., [Ca^2+^/CaM-independent]/[Ca^2+^/CaM-stimulated] activity) was measured in separate soluble kinase assays using the high-affinity CaMKII substrate AC-2. Autonomous activity was measured immediately after the prereaction (pre-SPOTs) and following the SPOT reaction (post-SPOTs); mean ± s.e.m. (*n* = 3). (d) Ca^2+^/CaM-stimulated SPOT substrate phosphorylation for various T^287^ autophosphorylation conditions; mean ± s.d. (*n* = 3). Results represent one-way ANOVA of log-normalized data with Holm-Šídák posttest compared to 0% autonomy. ^#^*P* < 0.05; ^##^*P* < 0.01; ^###^*P* < 0.001.

CaMKII_m_ autophosphorylation at T^287^ is a well-controlled, second-order reaction under saturating Ca^2+^/CaM conditions [15]. Like with CaMKII_holo_^+P^, the percentage of autophosphorylated subunits can be detected using the high-affinity substrate AC-2 (Figure 5(a)) which exhibits a 1 : 1 correlation in the number of T^287^ autophosphorylated subunits and maximal autonomous activity ([Ca^2+^/CaM-independent]/[Ca^2+^/CaM-stimulated]) [45]. Therefore, we generated populations of CaMKII_m_ containing varying ratios of CaMKII_m_^−P^ and CaMKII_m_^+P^ using temperature and time to vary the extent of autophosphorylated subunits (see Materials and Methods) (Figures 5(a) and 5(b)). Importantly, for each condition (i.e., extent of autophosphorylated CaMKII), the number of CaMKII_m_^+P^ subunits generated in the prereaction does not change during our standard SPOT assays, as the level of autonomous activity measured in a solution assay using the AC-2 substrate is the same pre- and post-SPOTs (Figure 5(c)). We observed a direct relationship between the extent of substrate phosphorylation and the percentage of T^287^ autophosphorylated subunits in the SPOT assay (Figure 5(d)). However, intermediate- and low-affinity substrates (Figure 5(d)) displayed the greatest increases in their phosphorylation in response to T^287^ autophosphorylation. Interestingly, the phosphorylation profile of the non-T^287^ autophosphorylated CaMKII_m_ (Figure 5(b)) was highly similar to the minimally activated CaMKII_holo_ (one subunit/holoenzyme condition in Figure 3(b), suggesting that inactivation (via T^306^ capping) can allow holoenzymes with limited activatable subunits to behave as non-T^287^-autophosphorylated monomers. Overall, our data support the hypothesis that the extent of activation and T^287^ autophosphorylation within the CaMKII holoenzyme functions to broaden the substrate specificity of CaMKII.

## 3. Discussion

Like other multifunctional protein kinases, CaMKII exhibits broad substrate specificity, targeting substrates involved in carbohydrate, amino acid and lipid metabolism, neurotransmitter synthesis and release, ion channels, receptors, transcription and translation, cytoskeletal organization and dynamics, cell cycle control, and Ca^2+^ homoeostasis [8]. In the nervous system, the role of CaMKII in synaptic plasticity has been intensively studied from both its role as a structural protein and as kinase [4–7]. In fact, while a number of different proteins and effector pathways have been implicated in regulating LTP over the years, CaMKII is considered to be a key mediator of this plasticity [46]. CaMKII is best known for signaling in the postsynaptic neuron for regulating the macromolecular signaling complex known as the PSD, a cytoskeletal organelle important for postsynaptic function. CaMKII translocation to the PSD is thought to be a critical feature of its function [47–51], a process that presumably functions to sequester and localize CaMKII activated with postsynaptic activity. The NMDA-R complex is a critical targeting molecule for CaMKII localization to the PSD and a substrate [27]. CaMKII phosphorylation of S^1303^ on the GluN2B subunit of the NMDA-R [52] reduces desensitization of the channel and hypothesized to serve as a feed-forward mechanism to sustain Ca^2+^ entry during learning and memory [53]. In addition to NMDA-R targeting, densin-180 and alpha-actinin also serve to recruit CaMKII to the PSD, possibly to simultaneously interact with CaMKII via multivalent contacts to nucleate and create a defined signaling complex [54] for the >40 CaMKII substrates identified at the PSD [55–57]. Thus, CaMKII translocation to the PSD appears to involve complex interactions that function to restrict CaMKII signaling to active postsynaptic sites. However, once targeted to the PSD, it is unknown how different substrates are selected to create specific functional outputs, including opposing forms of synaptic plasticity LTP and LTD. Thus, the purpose of our study was to determine how graded activity and T^287^ autophosphorylation impact the output of CaMKII, substrate phosphorylation, and selectivity.

We reasoned that for graded levels of CaMKII activation to be important for its signaling, then there should be an intrinsic advantage for multiple subunits to be activated within the holoenzyme versus a similar catalytic level of individual or monomeric CaMKII molecules in phosphorylating PSD substrates. Our studies revealed that while we began our phosphorylation reactions with ~2-fold monomeric CaMKII activity (as measured via a soluble assay) compared to multimeric CaMKII, the native form displays a catalytic advantage at phosphorylating purified PSDs *in vitro*. Thus, while the early work by Cline and colleagues using viral expression of monomeric constitutively active CaMKII (T^286^D) [58–60] clearly shows that monomeric CaMKII modulates axonal growth, glutamatergic synapse maturation and stabilization of dendritic arbors, our work demonstrates that the native holoenzyme has an advantage over the monomer in PSD phosphorylation *in vitro*. We reasoned that the diffusion-restricted substrate environment like the PSD with many different CaMKII substrates (and targeting proteins) takes advantage of the multivalent structure of CaMKII [54], a process that could also take advantage of graded activity within the same CaMKII holoenzyme. Multivalent interactions between CaMKII and its targeting proteins and substrates [54] may contribute to the catalytic advantage observed for native CaMKII over monomeric CaMKII. Future studies are required to explore the interplay between autophosphorylation-dependent substrate gating and the contribution of known PSD targeting proteins (e.g., NR2B, actin, and actinin) in this enhanced PSD phosphorylation by autophosphorylated CaMKII.

We explored this idea directly using multisubstrate SPOT membranes with peptide substrates synthesized from well-known CaMKII substrates. The four model substrates we selected differed in affinity (GluN2B_S1303_ > Syntide > Vimentin_S83_ and GluA1_S849_), possessed divergent phosphorylation motifs, and have been previously characterized *in vivo* and *in vitro* as protein and peptide substrates. Unlike the other three substrates, the GluN2B_S1303_ is a classic example of a T-site-interacting substrate (i.e., mimics CaMKII's ARD) which allows for this substrate to form high-affinity stable interactions to influence targeting [42] and catalytic activity [41, 61]. We elected to use the multisubstrate SPOT array approach because like isolated PSDs, we can explore CaMKII phosphorylation of diverse substrates simultaneously in a diffusion-restricted environment; however, unlike the PSD, the SPOT arrays avoid the intrinsic complexity produced by endogenous CaMKII, scaffolding, and regulatory proteins, factors that can be envisioned to influence our enzymatic analysis of substrate specificity.

To create graded levels of CaMKII activity autophosphorylation, we considered the following. First, the intraholoenzyme-intermolecular T^287^ autophosphorylation reaction is rapid (i.e., seconds) under saturating Ca^2+^/CaM conditions [15]. Second, while limiting Ca^2+^/CaM used to restrict the activation process, it will ultimately produce a heterogeneous mixture of activation states in CaMKII (±T^287^ autophosphorylation, ±T^306^ autophosphorylation, and/or ±Ca^2+^/CaM activation) that will be difficult to interpret. Third, creating mixed holoenzymes of dead or T^287^A mutants in our insect expression system has an obvious limitation in that quantitatively controlling the mutant to wild-type subunit ratios within individual holoenzymes will be difficult. Fourth, inhibitory autophosphorylation is used by CaMKII *in vivo* to create “capped” or Ca^2+^/CaM-insensitive subunits [4]. Thus, we elected to create graded levels of CaMKII activation by using this intrinsic property of CaMKII whereby inhibitory (Ca^2+^/CaM-independent) basal autophosphorylation inactivates CaMKII subunits [19, 38–40]. We created holoenzyme populations with varying ratios of capped subunits (nonresponsive to Ca^2+^/CaM and inactive) by incubating naïve multimeric CaMKII with Mg^2+^/ATP. This intramolecular subunit reaction [62] occurs in the absence of Ca^2+^/CaM as the autophosphorylation event leading to capping occurs within the CaM-binding domain (T^306^ in *δ*/*β*/*γ* CaMKII and T^305^ in *α* CaMKII) [38]. Using this strategy, we created CaMKII holoenzymes with graded levels of activity in the presence of saturating Ca^2+^/CaM to reveal dramatic shifts in the substrate specificity of the kinase. Higher affinity substrates appear to be preferentially phosphorylated when limited subunits are available for activation per holoenzyme (1/14). However, intermediate and weak substrates become utilized as multiple subunits are available for activation within the holoenzyme (5/15 and 14/14). This analysis requires the assumption that the capping protocol limiting subunit availability to Ca^2+^/CaM is stochastic at the microscopic level (i.e., individual subunit). The substrate phosphorylation profile for the CaMKII multimer (1/14) looks very similar to monomeric CaMKII in the absence of T^287^ autophosphorylation, suggesting that at the macroscopic level (bulk holoenzymes in solution), the theoretical 1/14 condition functionally approximates a single non-T^287^ autophosphorylated state. While we did have success in creating CaMKII with graded levels of activity (under saturating Ca^2+^/CaM), this protocol does not have sufficient control to separate different activation states (± T^287^ autophosphorylation in the capped CaMKII holoenzymes). Therefore, we employed monomeric CaMKII to explore the specific question of whether T^287^ autophosphorylation impacts substrate selectivity.

The role of autophosphorylation in regulating substrate accessibility was examined more recently and found to have mild [32] or no effect [33] on substrate phosphorylation. While differences in our conclusions may be attributable to different species of CaMKII (human versus rat) and isoforms (*α* versus *δ*), we do not favor these possibilities as the catalytic domain of all four CaMKII isoforms is highly conserved [9]. Historically, early structure-function studies demonstrated a lag in substrate catalysis that was relieved by CaMKII autophosphorylation [63]. Our substrate reactions used preautophosphorylated CaMKII and sufficiently dilute monomeric CaMKII in order to minimize this caveat. Our studies with monomer and native multimeric CaMKII indicate that graded levels of activation and/or T^287^ autophosphorylation are important determinants of intermediate and weak substrate phosphorylation. While high-affinity substrate phosphorylation is also enhanced by T^287^ autophosphorylation in CaMKII_m_^+P^, the effect was not seen in native CaMKII due to avidity effects. Whether this CaMKII_holo_^+P^ phenomenon is simply an artifact of peptide density generated in our SPOT membranes or operates in complex substrate environments like the PSD requires additional studies. However, our studies suggest that high-affinity substrates do not have the same requirement for T^287^ autophosphorylation as intermediate- or low-affinity substrates. In total, our data consistent with PSD substrates are tuned for phosphorylation by the multivalent structure of CaMKII and T^287^ autophosphorylation specifically broadens substrate specificity by enhancing intermediate and weak substrate utilization.

## 4. Conclusions

The unique structure and autoregulation make CaMKII an ideal sensor to encode Ca^2+^-spike frequency into graded levels of activity and T^287^ autophosphorylation within the holoenzyme. While this process is influenced by Ca^2+^-spike duration and amplitude [20] as well as phosphatase activity [21], alternative spliced linker length and CaM availability [22, 23], how the number of activated subunits and/or extent of T^287^ autophosphorylation elicits different functional outputs is unknown. Our studies indicate that the activation state of CaMKII, and specifically T^287^ autophosphorylation, is critical drivers of substrate selectivity. Furthermore, our studies suggest a mechanistic explanation for how CaMKII signaling could regulate even opposing functional outputs like LTP and LTD via alterations in substrate selectivity. Future studies are required to determine if graded activity and autophosphorylation operates within complex heterogeneous compartments like the PSD. Furthermore, while our highly reduced biochemical studies suggest that the substrate gating effect of T^287^ autophosphorylation is an intrinsic property of the catalytic domain (and independent of the holoenzyme), further studies are required to illuminate how this mechanism differentially regulates substrate accessibility.

## 5. Materials and Methods

### 5.1. Expression and Purification of CaMKII and Calmodulin

Recombinant human CaMKII*δ* (NCBI RefSeq: NP_742113.1) with an N-terminal 6xHN tag was integrated into a baculoviral construct (BacPAK9-6xHN), amplified in Sf9 insect cells (expression systems), and expressed in Hi5 (*T. ni;* (Expression Systems)) insect cells [64]. Site-directed mutagenesis was used to generate monomeric hCaMKII*δ*_1–317_ (i.e., CaMKII_m_) (by truncation through the addition of stop codon at aa318). Kinases were purified under reducing conditions by affinity chromatography (NiNTA resin) followed by size exclusion chromatography (Sephacryl S-400 [holoenzyme] or S-300 [CaMKII_m_]) using an Äkta Purifier (Amersham). SDS-PAGE of the purified proteins revealed a single band with purities > 98%. Recombinant calmodulin was expressed and purified in E.coli as described previously [12, 65] via boiling, ammonium sulfate precipitation, and phenyl-sepharose affinity chromatography.

### 5.2. Postsynaptic Density Purification

PSDs were acquired from male/female adult rats (Sprague-Dawley) using previously defined procedures involving differential centrifugation with various sucrose gradients followed by Triton X-100 extraction and collection of the Triton-insoluble fraction [34, 35]. Several steps were taken to limit and reduce the Ca^2+^/CaM-stimulated and autonomous activity of endogenous CaMKII within the PSDs. Rat brains were extracted and snap frozen in liquid nitrogen within 90 secs after sacrificing the animal, as CaMKII is known to activate and translocate to the PSDs within minutes in response to cell death [36].

### 5.3. Postsynaptic Density Phosphorylation

The PSDs were dephosphorylated to prevent autonomous activity of endogenous CaMKII (resulting from prior T^287^ autophosphorylation), as well as to reduce the phosphorylation state of endogenous substrates. This was accomplished with 1.5 *μ*M PP1*α* (6xHis purified) overnight at 4°C or at RT for 2 hours with shaking in the presence of 50 mM HEPES pH 7.4, 100 mM NaCl, 1 mM MnCl_2_, 0.015 Brij 35, 2.5 mM DTT, 10 *μ*M KN-93, and 2x calbiochem protease inhibitor cocktail set V. Phosphorylation reactions were carried out on dephosphorylated PSDs in the presence of 2x phosphatase inhibitor cocktail (Calbiochem) as well as the small molecule CaMKII inhibitor KN93 which only inhibits the autoinhibited kinase (naïve). PSD phosphorylation reactions were carried out on ~50 *μ*g of dephosphorylated PSDs (see above) in the presence of 20 mM HEPES pH 7.4, 100 mM NaCl, 10 mM MgCl_2_, 0.5 mM CaCl_2_, 5 *μ*M CaM, 100 *μ*M cold ATP, 120 *μ*Ci/ml [*γ*-^32^P]-ATP, and 350 nM CaMKII (per subunit). For reactions involving T^287^ autophosphorylated CaMKII, a prereaction was performed in the presence of 20 mM HEPES pH 7.4, 100 mM NaCl, 10 mM MgCl_2_, 0.5 mM CaCl_2_, 5 *μ*M CaM, 500 *μ*M ATP*γ*S, and 3.5 *μ*M kinase for 10 min at 30°C (for monomer) or on ice (for the holoenzyme). PSD phosphorylation reactions (60 *μ*l total) were incubated at room temperature for 4 min unless otherwise noted. Reactions were terminated with the addition of 500 *μ*l of 100 mM sodium phosphate pH 7.0, 1 M NaCl, and 10 mM EDTA) followed by centrifugation to obtain a PSD pellet. Pellet was three times in the same termination buffer. The PSD pellet was resuspended in 2x LDS sample buffer and resuspended using a horn sonicator. SDS-PAGE was performed followed by Coomassie staining and drying of the gel. Total protein was assessed by densitometry of the Coomassie signal while the extent of radioactive phosphate incorporation was quantified using a Fujifilm phosphorimager and profiles measured as photostimulated luminescence (PSL/mm^2^). The densitometric intensity data was converted to area under the curve (AUC) to determine to protein or total phosphorylation.

### 5.4. Soluble Peptide Assays

Soluble peptide substrates (generally 15mers) were obtained (Biopeptide Co. Inc.) and utilized in standard radioactive activity assays. In typical Ca^2+^/CaM-dependent reactions, 50-200 *μ*M substrate was used in the presence of 50 mM HEPES pH 7.4, 100 mM NaCl, 10 mM MgCl_2_, 0.2 mM CaCl_2_, 1 *μ*M CaM, 100 *μ*M cold ATP, 60 *μ*Ci/ml [*γ*-^32^P] ATP, and 5–10 nM CaMKII (per subunit). In Ca^2+^/CaM-independent reactions, 5 mM EGTA (or BAPTA) was used to chelate free Ca^2+^. This constitutive (i.e., autonomous) activity in the absence of Ca^2+^ was expressed, a percentage of the Ca^2+^/CaM-dependent activity. Reactions were carried out at 30°C for 1 minute and quenched by spotting onto Whatman Grade P81 ion exchange chromatography paper (Whatman, GE Healthcare, Piscataway, NJ). The filter papers were washed with 75 mM phosphoric acid 3 times for 5 minutes each and subsequently quantified in a scintillation counter (Beckman) via the Cerenkov counting method [66, 67]. AC-2 (KKALRRQEtVDAL), a CaMKII substrate derived from the CaMKII T^287^ autophosphorylation site was used for standard soluble peptide substrate reactions as described previously [64, 68].

### 5.5. Holoenzyme Inactivation

CaMKII_holo_ (600 nM) was exposed to inhibitory autophosphorylation (T^306/7^ capping) for 0, 5, or 15 minutes in the presence of 50 mM HEPES pH 7.4, 100 mM NaCl, 10 mM MgCl_2_, and 500 *μ*M ATP. Inactivated states of CaMKII were then diluted to 60 nM for SPOT substrate phosphorylation reactions (described below). For all states of preinactivation (0, 5, 8, or 15 min), the SPOT phosphorylation was normalized to the number of active subunits per holoenzyme (assuming each holoenzyme is tetradecameric). The number of active subunits per holoenzyme was assessed by measuring the Ca^2+^/CaM-dependent activity of each inactivation condition in standard soluble peptide substrate phosphorylation assays using AC-2 peptide. These activity assays were performed at the beginning and end (4 min) of the SPOT phosphorylation assay, their values averaged together. The activity from naïve CaMKII_holo_ (i.e., 0 min preinactivation) was considered to maximal (i.e., all 14 subunits activated) and was thus used to calculate the average enzymatic activity per subunit.

### 5.6. Preautophosphorylation Reactions

For reactions involving T^287^ autophosphorylated CaMKII, a prereaction was performed in the presence of 20 mM HEPES pH 7.4, 100 mM NaCl, 10 mM MgCl_2_, 0.5 mM CaCl_2_, 5 *μ*M CaM, 500 *μ*M cold ATP, and 500 nM kinase for 10 minutes on ice. To achieve varying numbers/percentages of T^287^ autophosphorylated subunits (%AutoP), similar prereactions were used with 50 nM kinase incubation times of 0, 5, or 20 minutes on ice, or 10 minutes at 30°C (Figure 5; preconditions 1–4, resp.).

### 5.7. *In Vitro* SPOT Phosphorylation Assay

Peptides (generally 15mers) were synthesized using the SPOT method [69, 70] onto *β*-alanine derivatized cellulose membranes via a MultiPep synthesizer (Intavis AG, Cologne, Germany). The peptide blots were blocked with 5% BSA in 50 mM HEPES pH 7.4, 100 mM NaCl, 1 mM EDTA, and 0.02% NP-40 for 30 minutes followed by three washes in 100 mM Tris–HCl pH 7.4. Membranes were subjected to a kinase phosphorylation assay in the presence of 50 mM HEPES pH 7.4, 100 mM NaCl, 10 mM MgCl_2_, 0.2 mM CaCl_2_, 1 *μ*M CaM, 0.2 mg/ml BSA, 1 mM DTT, 100 *μ*M cold ATP, 6–12 *μ*Ci/ml (*γ*-^32^P) ATP, and 5–10 nM CaMKII (per subunit). The reactions were incubated at room temperature for 4 minutes unless otherwise noted, terminated with three washes (100 mM sodium phosphate pH 7.0, 1 M NaCl, and 10 mM EDTA), and dried. The extent of radioactive phosphate incorporation was quantified using a Fujifilm phosphoimager and expressed as photostimulated luminescence (PSL/mm^2^) for a 1.5 mm × 1.5 mm circle centered on each spot. Three to four replicate spots were averaged for each peptide for a given condition with error bars indicating s.d., unless otherwise noted. GluN2B_S1303_ = RNKLRRQHsYDTFVD; Syntide = PLARTLsVAGLPGKK; GluA1_S849_ = GFCLIPQQsINEAIR; Vimentin_S83_ = PGVRLLQDsVDFSLA.

### 5.8. Data and Statistical Analysis

One-way analysis of variance (ANOVA) with subsequent Holm-Šídák posttest was performed on log transformed to account for mean-dependent variances, compared to control (#). Statistical analyses were performed using SigmaPlot 12.5. Statistical significance is denoted by the number of symbols for different *P* values (e.g., ^#^*P* < 0.05, ^##^*P* < 0.01, and ^###^*P* < 0.001).

## Abbreviations

CaMKII: Calcium/calmodulin-dependent protein kinase II
AUC: Area under the curve
PSD: Postsynaptic density.
